# Supplementary material for: Deciphering how early life adiposity influences breast cancer risk using Mendelian randomization
Source: Commun Biol. 2022 Apr 8;5:337. doi: 10.1038/s42003-022-03272-5 (PMC8993830; doi:10.1038/s42003-022-03272-5)
Supplement: Supplementary file 2 — Supplementary Information [file 42003_2022_3272_MOESM2_ESM.pdf]

Supplementary Information

For the article “Deciphering how early life adiposity influences breast cancer risk using Mendelian randomization“, Vabistsevits *et al* 2022

Supplementary Note 1: Mediator prioritisation chart and Supplementary Table 1

Supplementary Note 2: Sensitivity analysis results

Supplementary Note 3: Split sample results

Supplementary Note 4: Additional MR analyses
- Total vs direct effect
- MVMR with extra traits

Supplementary Note 5: Mediation analysis details
- Mediation methods
- Standard error estimation
- Mediation analysis full results
- Simulation analysis for selecting the approach for mediation analysis
- Proportion mediated calculation

Supplementary Note 6: GWAS validation with LD Score Regression: Supplementary Table 2

Supplementary Note 1. Mediator prioritisation

Mediator prioritisation can be visualised in two ways – decision tree (Supplementary Figure 1) and decision table (Supplementary Table 1). Both show the conditions/performance that a mediator has to meet/show to be 'qualified' to be analysed in further steps that require the data to be robust. The evidence of effect implies the standard 95% CIs not overlapping the null, and the adequate performance in sensitivity analyses implies a) overall agreement between the main method (IVW) and MR-Egger and weighted median methods results and b) acceptable values of the estimated Egger intercept and Q-stat (see Methods and Supplementary Note 2 below).

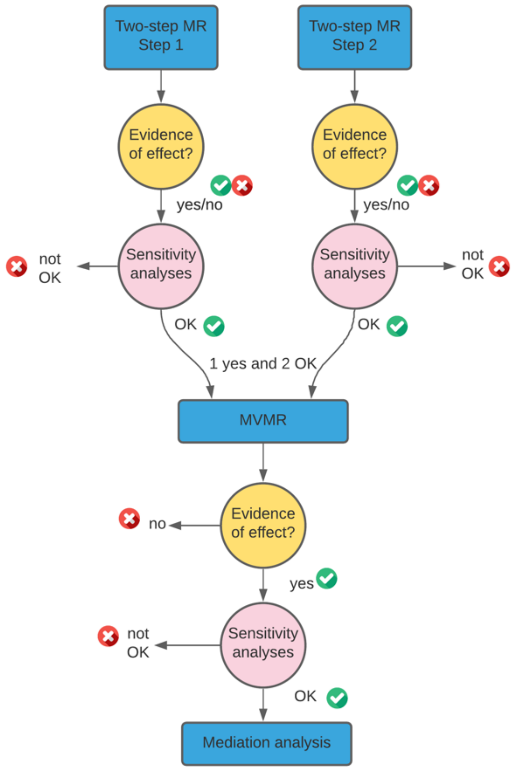

Supplementary Figure 1. Mediator prioritisation decision tree. *For MVMR*: at least one step from two-step MR has to have evidence of effect, and both have to show adequate sensitivity tests results. *For mediation*: MVMR effect and satisfactory F-statistics (sensitivity analysis).

Supplementary Table 1. Mediator prioritisation decision table. "Effect" is evidence of a causal effect in the analysis (1 - present, 0 - not); "Sensitivity/F-stats" indicates consistent and satisfactory performance in the sensitivity analyses (1 – yes, 0 – no); "Do MVMR/mediation?" indicates whether the test was selected/prioritised to be done based on effect/sensitivity. *For MVMR*: at least one step has 'effect', and both show adequate sensitivity tests results. *For mediation*: MVMR effect and satisfactory F-statistics (>10).

	Step 1 effect	Step1 sensitivity	Step 2 effect	Step2 sensitivity	Do MVMR?	MVMR effect	F-stats	Do mediation?
IGF-1	1	1	1	1	yes	1	1	yes
SHBG	1	1	0	1	yes	0	1	no
Oestradiol	1	0	0	0	no			no
Testosterone (bio)	1	1	1	1	yes	1	1	yes
Testosterone(free)	1	1	1	1	yes	1	1	yes
Testosterone(total)	0	1	1	1	yes	1	1	yes
Age at first birth	1	1	0	0	no			no
Age at menarche	1	1	0	1	yes	1	1	yes
Age at menopause	0	1	1	1	yes	1	1	yes
Number of births	0	1	0	0	no			no
Fasting glucose	0	1	0	1	no			no
Fasting insulin	1	1	0	0	no			no
Hb1ac	1	1	0	1	yes	0	1	no
HOMA-B	1	1	0	0	no			no

Supplementary Note 2. Sensitivity analysis results

The sensitivity analyses of step 1 of two-step MR analyses (effect of childhood body size on mediators) showed consistent results across all mediator groups (Supplementary Data 11). The direction of estimates was the same for all tested mediators, and the magnitude of the estimate was comparable across three MR methods, except for a higher MR-Egger effect observed for SHBG. The MR-Egger intercepts were close to zero (< +/-0.003) with large p-values (> 0.2), indicating little evidence of directional pleiotropy, except for fasting insulin, which had an intercept p-value of -0.0045 with p-value 0.01. Cochran's Q values were large with small p-values (<10e-6) for hormones and reproductive traits, suggesting the presence of heterogeneity. Considerably smaller Q-values and larger corresponding p-values were observed for all glycaemic traits and oestradiol.

The sensitivity analyses of step 2 of two-step MR analyses (effect of mediators on breast cancer) showed less robust results for some mediator groups (Supplementary Data 12). Among the hormones, the direction of effect was consistent across all three MR methods, and the Egger intercept was small (close to zero) with a large p-value. Cochran's Q p-values were also small, indicating little evidence for heterogeneity in the hormones group. The exception was oestradiol, for which only one genome-wide significant SNP was available, meaning only a single Wald ratio test could be performed, and its effect has to be interpreted with caution. For reproductive traits, the direction of the effects was consistent, and the intercepts were sufficiently close to zero, but MR-Egger produced wider confidence intervals in all cases. None of the glycaemic traits showed evidence of the effect in IVW, and horizontal pleiotropy detected with other methods suggests a likely bias of the estimates towards the null. For fasting insulin, MR-Egger analysis showed a strong effect in the opposite direction (observed on two out of three data sources, although based on < 5 SNPs), with Egger intercept for the main data source being away from zero (0.055) with a p-value of 0.08. Similarly, HOMA-B had a limited number of instruments, and sensitivity tests highlighted its potentially biased and inconclusive results. Fasting glucose and Hb1ac performed adequately in the sensitivity tests. Finally, in the physical traits group, as only a limited number of instruments was available, as expected, most sensitivity analyses detected problems.

Supplementary Note 3. Additional MVMR analyses

Total vs direct effect of selected mediators on breast cancer

Supplementary Figure 2 shows the total and direct effect of mediators on breast cancer. For all mediators (except age at menarche), the effects are very similar. In the case of age at menarche, in univariable MR, there was little evidence of total effect, while in MVMR, the direct effect (i.e. effect accounted for childhood body size) point estimate was notably more negative with CIs showing the evidence of this effect. The estimates are available in Supplementary Data 1 and 5.

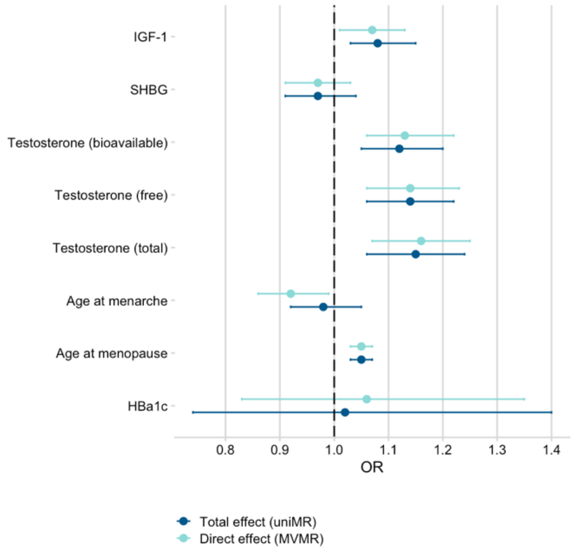

Supplementary Figure 2. Comparison of total and direct effects of a subset of mediators on breast cancer. Total effects were estimated in univariable MR (step 2), and direct effect in MVMR with childhood body size effect taken into account. Bars indicate 95% confidence intervals around the point estimates from IVW analyses.

MVMR with additional variables

The main MVMR analysis in this project aimed to separate the effects of childhood body size and a selected mediator. However, it was also important to consider if any effect can be explained by other related anthropometric measures. Therefore, we carried out additional MVMR analyses where we added adult BMI and childhood height as the additional variable in the MVMR model (two separate analyses). Supplementary Figure 3 shows the direct effects of mediators on breast cancer from the three models.

When accounted for the effect of adult body size, the mediator effect estimates were not affected considerably compared to childhood body size. However, accounting for childhood height reduced the direct estimates for most mediators and attenuated IGF-1 and age at menarche effects to overlap the null. The estimates are available in Supplementary Data 5, 9, 10. The sensitivity tests results are in Supplementary Data 13-15.

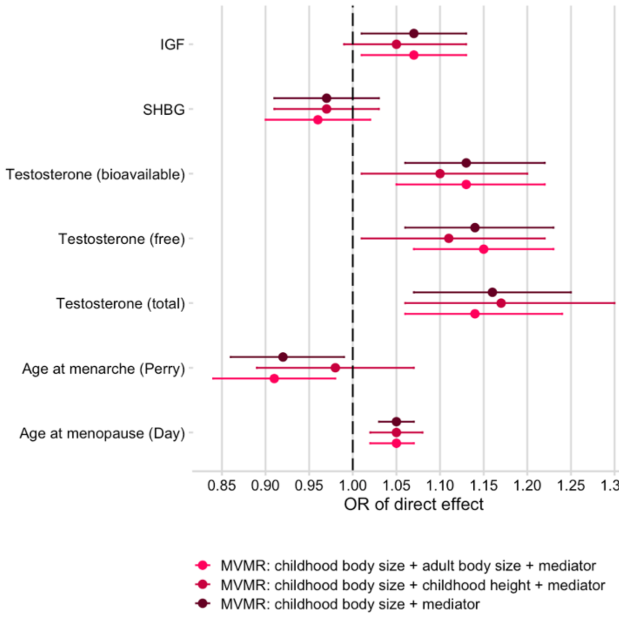

Supplementary Figure 3. Comparison of the direct effect of mediators on breast cancer while accounting for childhood body size and other traits. The MVMR models account for (1) childhood body size only (main MVMR analysis), (2) childhood body size and adult body size, and (3) childhood body size and childhood height. Bars indicate 95% confidence intervals around the direct effect point estimates from MVMR analyses.

Supplementary Note 4. Split-sample results

Supplementary Figure 4 shows the MR results from full-sample and split-sample analyses of childhood body size (exposure) and selected hormones and reproductive traits (outcomes). For hormones, the effect size and CIs were very similar between the two analyses. In the split-sample analysis of reproductive traits, we observed larger effect sizes with wider CIs on age at menarche, age at first birth, and age at menarche. Overall, the sample overlap in the full-sample analysis does not seem to be upwardly biasing estimates, on the contrary, it seems to produce more conservative estimates. Therefore, we conclude that, in this study, using the same sample for both exposure and outcome has not introduced any substantial bias in the effect estimates.

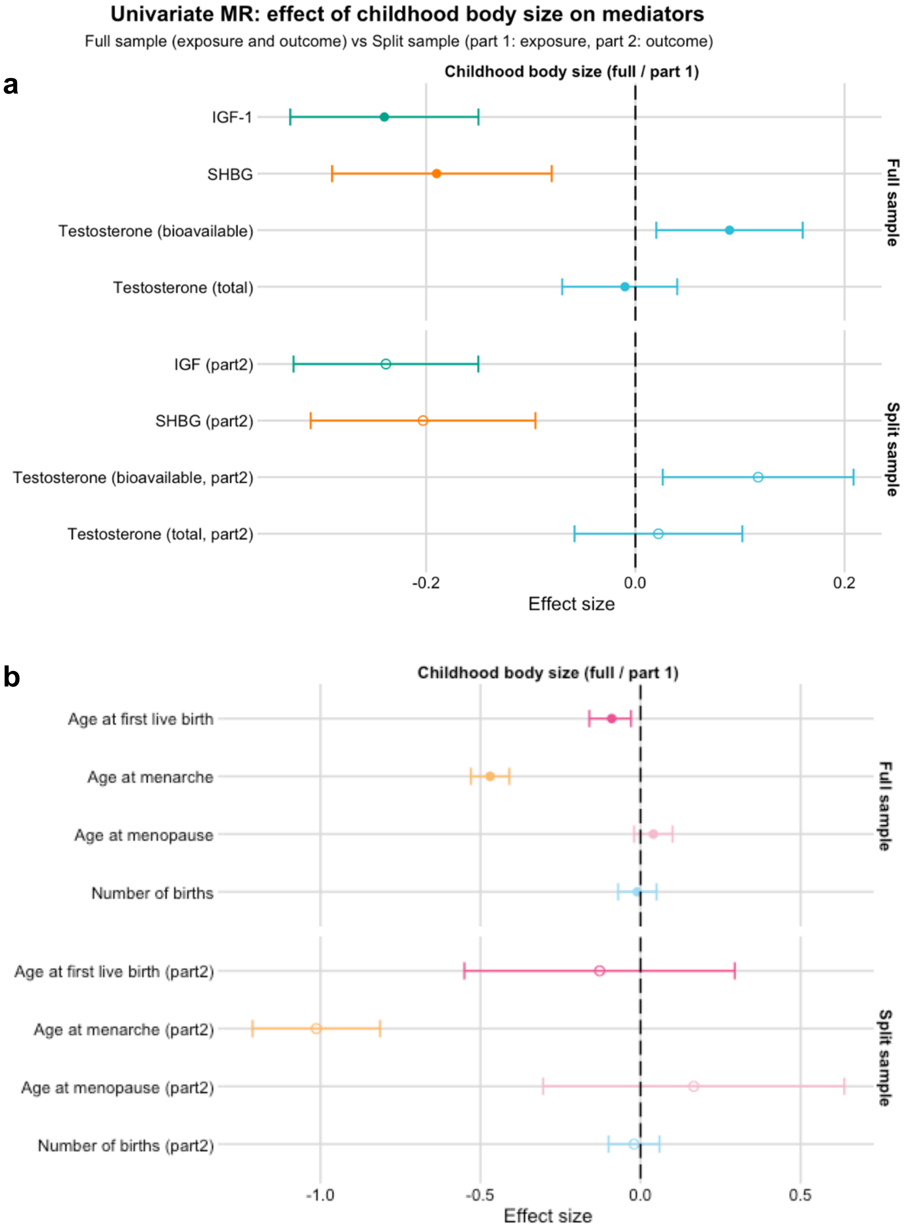

Supplementary Figure 4. Comparison of childhood body size effect estimates on (a) hormones and (b) reproductive traits from the full- and split-sample analyses. In the full-sample analysis, the entire UK Biobank cohort was used to extract exposure instruments (childhood body size), and it was used as the other GWAS (hormones / reproductive traits). In the split-sample analysis, the cohort was randomly split into parts, and part 1 was used to extract exposure instruments, while part 2 was used as the outcome GWAS. Bars indicate 95% confidence intervals around the point estimates from split-sample IVW analyses.

Supplementary Note 5. Mediation analysis details

Mediation methods

As outlined in Supplementary Figure 5, in Difference method, to estimate the indirect effect, we subtract the direct effect of exposure on the outcome from MVMR (model with the mediator of interest) from the total effect of exposure on the outcome (univariable MR)[1]. In Product method (also known as 'product of coefficients'), we multiply the results from two steps of two-step MR analysis (i.e., the effect of exposure on the mediator and the effect of the mediator on the outcome) to get the indirect effect[2,3]. It is sometimes recommended to replace the second term with the direct effect of the mediator on the outcome from MVMR analysis[4], but the results should be similar if there is sufficient instrument strength. In this paper, the direct effect was used in the analysis.

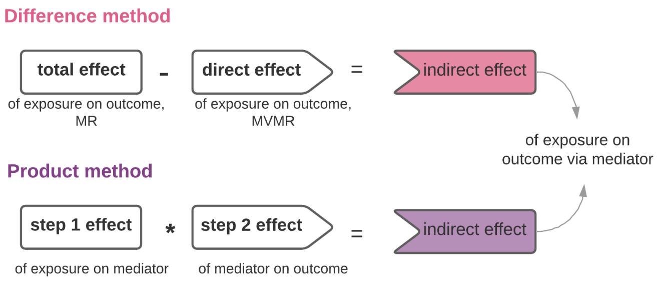

Supplementary Figure 5. Mediation analysis methods. *Difference method:* the indirect effect is estimated as a difference between the total and direct effect of exposure on the outcome, from univariable MR and multivariable MR, respectively. *Product method:* the indirect effect is estimated as the product of coefficients of the total effect of exposure on the mediator (step 1 of two-step MR) and the total effect of the mediator on the outcome (step 2 of two-step MR). The second term can be replaced by the direct effect of the mediator on the outcome from multivariable MR.

Standard error estimation methods

For the estimation of the confidence intervals (CIs) of the indirect effect, standard error (SE) has to be calculated. For each mediation method, there is a corresponding SE calculation method. To calculate SE of indirect effect from Difference method, 'Propagation of errors' (PoE) is used, as outlined in Burgess et al (2017)[1]. For Product method indirect effect SE calculation, we can use Delta method (also known as the Sobel test[5]) or a simplified version of PoE.

"Delta method" – can be applied to product method results

```
se.indirect = sqrt(a_beta^2 * b_se^2  +  b_beta^2 * a_se^2)
```

where

- `a_beta` and `a_se` are the total effect and SE from the step 1 MR analysis (exposure->mediator)
- `b_beta` and `b_se` are the total effect and SE from the step 2 MR analysis (mediator -> outcome), or the direct effect and SE from MVMR (mediator -> outcome)

"Propagation of errors method" – can be applied to both difference method and product method results

```
se.indirect = sqrt(se_total ^2 + se_direct^2)

se.indirect = sqrt(se_a^2 + se_b^2)
```

where

- `se_total` is the SE from the exposure -> outcome MR
- `se_direct` is the SE from exposure + mediator -> outcome MVMR (of exposure->outcome)

Mediation analysis full results

The indirect effect estimates calculated using Difference and Product methods were not consistent with one another in size and, in some cases, direction (Supplementary Figure 6, Supplementary Data 18). The confidence intervals based on SE calculated with Delta and PoE methods were also quite different, with PoE proving 10-fold magnitude SE, which resulted in wider confidence intervals. The inconsistent results made it difficult to draw any conclusions by evaluating both methods. Therefore, we carried out a simulation analysis (next section) to help us decide which combination of methods should be used for the mail analysis.

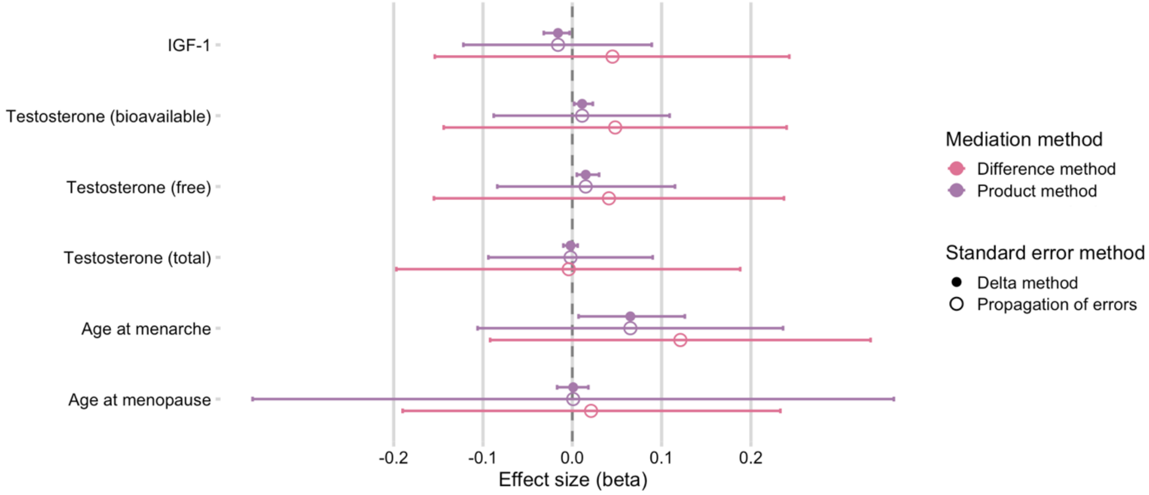

Supplementary Figure 6. Results of mediation analysis: indirect effect of childhood body size via mediators. The point estimates represent the indirect effect (beta) that childhood body size has on breast cancer risk via the specified mediator (left-hand side labels). Indirect effects were calculated using Difference method (pink) and Product method (purple). The standard error of beta (which is used in confidence intervals calculation) was estimated using Delta method (filled circle) and "propagation of errors" (empty circle); both methods were applied to Product method's beta. Bars indicate 95% confidence intervals around the point estimates.

Simulation analysis for selecting the approach for mediation analysis

The available methods for mediation analysis produced results with some differences both in the estimation of indirect effect beta and standard error (described in the previous section). To estimate which combination of methods should be used in our study, we carried out a simulation analysis.

Firstly, based on the results from two-step and MVMR analyses we generated a model to simulate the individual-level data. This data was used to create simulated summary statistics and then apply the IVW method (linear regression) to estimate the effects from the simulated data (exposure -> mediator, mediator -> outcome, exposure + mediator -> outcome). Separate models were set up to represent each mediator individually. For each mediator, we carried out 1000 iterations of such simulations. In each iteration, we estimated the indirect effect using Difference and Product methods from the simulated data and also calculated the corresponding SE using Delta and PoE methods.

Finally, we reviewed the distribution of indirect point estimates (from two mediation methods), i.e. we calculated the standard deviation of the estimated betas across each iteration, and compared it with the average of the standard errors calculated by each method described above across all iterations, to assess the similarity across the pairs of methods. The results are available in Supplementary Data 19.

Across all of the simulations, there was a high level of consistency between the SD of beta from Product method and the mean of SE from Delta method (e.g. 0.0019 for IGF1). This similarity was observed for all mediators in the analysis. The other combination of methods (involving PoE method for SE estimation) produced much higher SE than the true value from the simulation. As a result, the combination of Product and Delta methods was chosen for the main analysis.
The simulation implementation is available here: https://github.com/mvab/simulation_for_MR_mediation

Proportion mediated calculation

The proportion of exposure effect mediated via a mediator is calculated as mediator indirect effect divided by the total effect beta[4]. For IGF-1, the proportion mediated was calculated as follows:

-0.016 / -0.411 = 0.039, i.e.

Product method-estimated indirect beta (Supplementary Data 18) *divided by* the total effect beta (Supplementary Data 6)

Supplementary Note 6. GWAS validation with LD Score Regression

To validate the consistency of genetic instruments from GWAS generated in UK Biobank as a part of this study (IGF-1, SHBG, testosterone) with the published analyses of the same traits, we performed LD Score Regression[6] (Supplementary Table 2).

For the analysis, we used the data from the studies by Ruth *et al* 2020[7] (total and bioavailable testosterone, SHBG – data available from GWAS Catalog) and Murphy *et al* 2019[8] (IGF-1 – data from Neale Lab: www.nealelab.is/uk-biobank).

The *rg* score (genetic correlation) between our study data and the published data is very close to 1, i.e. the datasets are ideally correlated, despite slight differences in sample sizes and covariates.

Supplementary Table 2. LD Score Regression results between GWAS generated in this study and published studies

	rg	lo_ci	up_ci	se	pval
**IGF-1**	1.00	0.99	1.00	0.0025	< 1e-300
**SHBG**	1.00	0.99	1.01	0.0046	< 1e-300
**Bioavailable testosterone**	1.00	0.99	1.01	0.0031	< 1e-300
**Total testosterone**	0.99	0.98	1.01	0.0064	< 1e-300

Supplementary references

1. Burgess, S. *et al.* Dissecting causal pathways using mendelian randomization with summarized genetic data: Application to age at menarche and risk of breast cancer. *Genetics* **207**, 481–487 (2017).
2. Burgess, S., Daniel, R. M., Butterworth, A. S. & Thompson, S. G. Network Mendelian randomization: Using genetic variants as instrumental variables to investigate mediation in causal pathways. *International Journal of Epidemiology* **44**, 484–495 (2015).
3. Relton, C. L. & Davey Smith, G. Two-step epigenetic mendelian randomization: A strategy for establishing the causal role of epigenetic processes in pathways to disease. *International Journal of Epidemiology* **41**, 161–176 (2012).
4. Carter, A. R. *et al.* Mendelian randomisation for mediation analysis: Current methods and challenges for implementation. *European Journal of Epidemiology* (2021) doi:10.1101/835819.
5. Sobel, M. E. Asymptotic Confidence Intervals for Indirect Effects in Structural Equation Models. *Sociological Methodology* **13**, 290–312 (1982).
6. Bulik-Sullivan, B. *et al.* LD Score regression distinguishes confounding from polygenicity in genome-wide association studies. *Nature Genetics 2015 47:3* **47**, 291–295 (2015).
7. Ruth, K. S. *et al.* Using human genetics to understand the disease impacts of testosterone in men and women. *Nature Medicine* **26**, 252–258 (2020).
8. Murphy, N. *et al.* Insulin-like growth factor-1, insulin-like growth factor-binding protein-3, and breast cancer risk: observational and Mendelian randomization analyses with ~430 000 women. *Annals of Oncology* **31**, 641–649 (2020).
