# Supplementary material for: Deciphering how early life adiposity influences breast cancer risk using Mendelian randomization
Source: Commun Biol. 2022 Apr 8;5:337. doi: 10.1038/s42003-022-03272-5 (PMC8993830; doi:10.1038/s42003-022-03272-5)
Supplement: Supplementary file 3 — Description of Additional Supplementary Files [file 42003_2022_3272_MOESM3_ESM.pdf]

Description of Additional Supplementary Files

File name: Supplementary Data 1-20

Description: Results tables from two-step MR, MVMR, sensitivity analysis, mediation analysis, simulation analysis, and source data for the figures.
