# Supplementary material for: Deciphering how early life adiposity influences breast cancer risk using Mendelian randomization
Source: Commun Biol. 2022 Apr 8;5:337. doi: 10.1038/s42003-022-03272-5 (PMC8993830; doi:10.1038/s42003-022-03272-5)
Supplement: Supplementary file 5 — Reporting Summary [file 42003_2022_3272_MOESM5_ESM.pdf]

nature portfolio

Corresponding author(s): Marina Vabistsevits

Last updated by author(s): Feb 17, 2022

Reporting Summary

Nature Portfolio wishes to improve the reproducibility of the work that we publish. This form provides structure for consistency and transparency in reporting. For further information on Nature Portfolio policies, see our Editorial Policies and the Editorial Policy Checklist.

Statistics

For all statistical analyses, confirm that the following items are present in the figure legend, table legend, main text, or Methods section.

n/a	Confirmed	
☐	☒	The exact sample size (*n*) for each experimental group/condition, given as a discrete number and unit of measurement
☐	☒	A statement on whether measurements were taken from distinct samples or whether the same sample was measured repeatedly
☐	☒	The statistical test(s) used AND whether they are one- or two-sided<br>*Only common tests should be described solely by name; describe more complex techniques in the Methods section.*
☐	☒	A description of all covariates tested
☐	☒	A description of any assumptions or corrections, such as tests of normality and adjustment for multiple comparisons
☐	☒	A full description of the statistical parameters including central tendency (e.g. means) or other basic estimates (e.g. regression coefficient) AND variation (e.g. standard deviation) or associated estimates of uncertainty (e.g. confidence intervals)
☐	☒	For null hypothesis testing, the test statistic (e.g. *F*, *t*, *r*) with confidence intervals, effect sizes, degrees of freedom and *P* value noted<br>*Give P values as exact values whenever suitable.*
☒	☐	For Bayesian analysis, information on the choice of priors and Markov chain Monte Carlo settings
☐	☒	For hierarchical and complex designs, identification of the appropriate level for tests and full reporting of outcomes
☐	☒	Estimates of effect sizes (e.g. Cohen's *d*, Pearson's *r*), indicating how they were calculated

Our web collection on statistics for biologists contains articles on many of the points above.

Software and code

Policy information about availability of computer code

Data collection	All code is available at https://github.com/mvab/mendelian-randomization-breast-cancer
Data analysis	All code is available at https://github.com/mvab/mendelian-randomization-breast-cancer

For manuscripts utilizing custom algorithms or software that are central to the research but not yet described in published literature, software must be made available to editors and reviewers. We strongly encourage code deposition in a community repository (e.g. GitHub). See the Nature Portfolio guidelines for submitting code & software for further information.

Data

Policy information about availability of data

All manuscripts must include a data availability statement. This statement should provide the following information, where applicable:

- Accession codes, unique identifiers, or web links for publicly available datasets
- A description of any restrictions on data availability
- For clinical datasets or third party data, please ensure that the statement adheres to our policy

The GWAS summary statistics generated in this study are available through the OpenGWAS platform (https://gwas.mrcieu.ac.uk/) upon publication. The other data sources used are listed in Table 1 of the manuscript.

Field-specific reporting

Please select the one below that is the best fit for your research. If you are not sure, read the appropriate sections before making your selection.

☒ Life sciences ☐ Behavioural & social sciences ☐ Ecological, evolutionary & environmental sciences

For a reference copy of the document with all sections, see nature.com/documents/nr-reporting-summary-flat.pdf

Life sciences study design

All studies must disclose on these points even when the disclosure is negative.

Sample size	The UK Biobank GWAS performed as a part of this study were done on female-only samples of the cohort and have slightly different sample sizes depending on the phenotype data availability: childhood body size (246,511), IGF1 (246,284), oestradiol (53,491), SHBG (222,491), testosterone (186,700). The sample sizes of other traits' GWAS are available in Table 1 of the manuscript; those sample sizes were pre-defined as we used publicly available GWAS summary statistics.
Data exclusions	UK Biobank male samples were excluded as we were interested in sex-specific effects of hormonal traits. Other exclusions included non-European and related individuals as a part of the standard UK Biobank data processing.
Replication	Replication of Mendelian Randomization analyses was performed if there was data available for the trait measured in different cohort.
Randomization	NA - randomization occurred naturally as genetic variants were the exposure.
Blinding	NA - genetic association testing does not require blinding.

Reporting for specific materials, systems and methods

We require information from authors about some types of materials, experimental systems and methods used in many studies. Here, indicate whether each material, system or method listed is relevant to your study. If you are not sure if a list item applies to your research, read the appropriate section before selecting a response.

Materials & experimental systems

n/a	Involved in the study	
☒	☐	Antibodies
☒	☐	Eukaryotic cell lines
☒	☐	Palaeontology and archaeology
☒	☐	Animals and other organisms
☐	☒	Human research participants
☒	☐	Clinical data
☒	☐	Dual use research of concern

Methods

n/a	Involved in the study	
☒	☐	ChIP-seq
☒	☐	Flow cytometry
☒	☐	MRI-based neuroimaging

Human research participants

Policy information about studies involving human research participants

Population characteristics	UK Biobank is an ongoing large-scale prospective cohort study with genetic and phenotypic data collected on approximately 500,000 individuals from across the UK, aged 40-69. Further details are available in Bycroft et al 2018 "The UK Biobank resource with deep phenotyping and genomic data"
Recruitment	UK Biobank participants were recruited between the years 2006 and 2010 from across the UK, aged 40-69 at recruitment. A rich variety of phenotypic and health-related information is available on each participant, including biological measurements, lifestyle indicators, biomarkers in blood and urine, and imaging of the body and brain. Further details are available in Bycroft et al 2018 "The UK Biobank resource with deep phenotyping and genomic data"
Ethics oversight	UK Biobank has received ethical approval from the UK National Health Service's National Research Ethics Service (ref 11/NW/0382). At recruitment, all participants gave informed consent to participate in the UK Biobank and be followed-up with. The analysis of other datasets was done on summary statistics and does not require additional ethical approval.

Note that full information on the approval of the study protocol must also be provided in the manuscript.
